# The Differential Interaction of *Brucella* and *Ochrobactrum* with Innate Immunity Reveals Traits Related to the Evolution of Stealthy Pathogens

**DOI:** 10.1371/journal.pone.0005893

**Published:** 2009-06-16

**Authors:** Elías Barquero-Calvo, Raquel Conde-Alvarez, Carlos Chacón-Díaz, Lucía Quesada-Lobo, Anna Martirosyan, Caterina Guzmán-Verri, Maite Iriarte, Mateja Mancek-Keber, Roman Jerala, Jean Pierre Gorvel, Ignacio Moriyón, Edgardo Moreno, Esteban Chaves-Olarte

**Affiliations:** 1 Programa de Investigación en Enfermedades Tropicales, Escuela de Medicina Veterinaria, Universidad Nacional, Heredia, Costa Rica; 2 Department of Microbiology, University of Navarra, Navarra, Spain; 3 Centro de Investigación en Enfermedades Tropicales, Facultad de Microbiología, Universidad de Costa Rica, San José, Costa Rica; 4 Centre d'Immunologie de Marseille-Luminy, Aix Marseille Université, Faculté de Sciences de Luminy, INSERM U631, CNRS UMR6102, Marseille, France; 5 Department of Biotechnology, National Institute of Chemistry, Hajdrihova, Ljubljana, Slovenia; University of California Merced, United States of America

## Abstract

**Background:**

During evolution, innate immunity has been tuned to recognize pathogen-associated molecular patterns. However, some α-*Proteobacteria* are stealthy intracellular pathogens not readily detected by this system. *Brucella* members follow this strategy and are highly virulent, but other *Brucellaceae* like *Ochrobactrum* are rhizosphere inhabitants and only opportunistic pathogens. To gain insight into the emergence of the stealthy strategy, we compared these two phylogenetically close but biologically divergent bacteria.

**Methodology/Principal Findings:**

In contrast to *Brucella abortus*, *Ochrobactrum anthropi* did not replicate within professional and non-professional phagocytes and, whereas neutrophils had a limited action on *B. abortus*, they were essential to control *O. anthropi* infections. *O. anthropi* triggered proinflammatory responses markedly lower than *Salmonella enterica* but higher than *B. abortus*. In macrophages and dendritic cells, the corresponding lipopolysaccharides reproduced these grades of activation, and binding of *O. anthropi* lipopolysaccharide to the TLR4 co-receptor MD-2 and NF-κB induction laid between those of *B. abortus* and enteric bacteria lipopolysaccharides. These differences correlate with reported variations in lipopolysaccharide core sugars, sensitivity to bactericidal peptides and outer membrane permeability.

**Conclusions/Significance:**

The results suggest that *Brucellaceae* ancestors carried molecules not readily recognized by innate immunity, so that non-drastic variations led to the emergence of stealthy intracellular parasites. They also suggest that some critical envelope properties, like selective permeability, are profoundly altered upon modification of pathogen-associated molecular patterns, and that this represents a further adaptation to the host. It is proposed that this adaptive trend is relevant in other intracellular α-*Proteobacteria* like *Bartonella*, *Rickettsia*, *Anaplasma*, *Ehrlichia* and *Wolbachia*.

## Introduction

The class α-*Proteobacteria* includes microorganisms capable of establishing close associations with eukaryotic cells in a wide range of lifestyles. Members of the genus *Agrobacterium* are pericellular to plant cells and induce tumors, whereas *Brucella*, *Bartonella*, *Phyllobacterium* and *Sinorhizobium* are facultative extracellular-intracellular bacteria that behave as pathogens or endosymbionts, and *Rickettsia*, *Anaplasma*, *Ehrlichia* and *Wolbachia* are obligate intracellular pathogens of mammals and arthropods [1], [2]. Remarkably, animal pathogens of this group have the ability to avoid immediate recognition by innate immunity, thus following a stealthy strategy of which *Brucella* can be considered as a model [3]. In contrast, some bacteria close to *Brucella* are free living environmental microorganisms like *Ochrobactrum*, *Daeguia*, *Crabtreella* and *Mycoplana*. Although these four genera are included in the *Brucellaceae* (http://www.bacterio.cict.fr/), only *Ochrobactrum* has been reported to display some degree of pathogenicity. *Ochrobactrum anthropi*, primarily a rhizosphere inhabitant, has been isolated from immunocompromised individuals or patients subjected to dialysis, catheterization, surgical interventions or transplantation [4], [5], [6], [7], [8], [9], [10], [11] and often shows a broad antibiotic resistance [12], [13]. *Ochrobactrum intermedium* is another opportunistic member of the genus. Interestingly, *Ochrobactrum* is the *Brucella*ceae member closest to *Brucella* according to several molecular markers and genome comparisons [14].

Owing to its close phylogenetic relatedness with the highly virulent *Brucella* [15], *Ochrobactrum* has received some attention. Phenotypic analysis reveals that *O. anthropi* displays envelope molecules known to be critical in *Brucella* virulence. They include phosphatidylcholine and a lipopolysaccharide (LPS) with a lipid A carrying very long chain fatty acids (VLCFA). Also, like other *Brucellaceae*, *O. anthropi* free lipids contain acyl chains with a higher number of carbons than those found in typical Gram negative bacteria. Along with the VLCFAs of lipid A, these structural features are thought to be relevant in the construction of a firm envelope which, in the case of *Brucella*, has been proposed to be important in pathogenicity [16], [17]. Despite these similarities, there are differences in important outer membrane properties. Most notably, while *Brucella* is highly permeable to hydrophobic substances and resistant to bactericidal cationic peptides, *Ochrobactrum* is impermeable and sensitive, albeit not to the same extent as typical Gram negative bacteria [18]. Interestingly, these differences have been correlated with some structural variations in the LPSs of these bacteria [18]. LPS typically bears a marked pathogen-associated molecular pattern (PAMP) and is thus a key target of innate immunity [19]. Indeed, the altered PAMP of *Brucella* LPS (*Ba*LPS) greatly contributes to the stealthy strategy and virulence of this pathogen [3], [20]. All this evidence leads to the hypothesis that the *Brucellaceae* ancestors carried such molecules that without extensive remodeling resulted in features adequate to evade innate immunity [3].

To examine this possibility, we compared *O. anthropi* with *Brucella abortus*. We found that these bacteria display a significant divergence in the interaction with host cells and the innate immune system, and traced some of the differences to changes in orthologs of key *Brucella* virulence determinants. The results not only help to understand the widely different degrees of pathogenicity of these bacteria but also how intracellular pathogens following a stealthy strategy may have emerged in the α-*Proteobacteria*.

## Results

### *O. anthropi* does not multiply intracellularly

**Figure 1:**
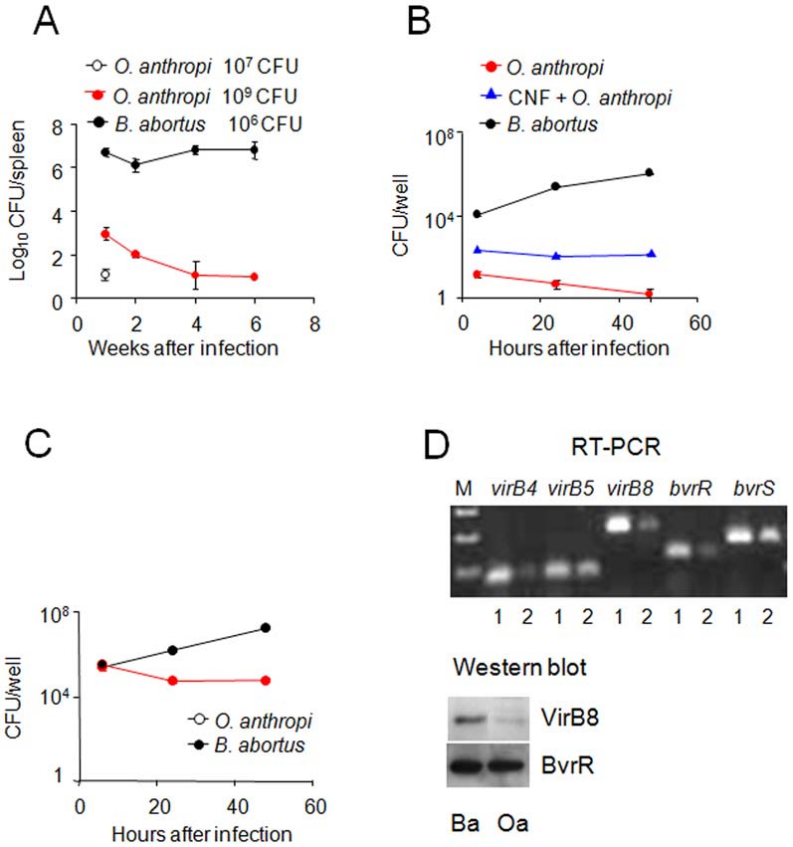
*O. anthropi* does not replicate in mice or in cells but expresses *Brucella*-associated virulence factors. (A) Groups of six mice were inoculated intraperitoneally with 10^7^ (white circles) or 10^9^ (red circles) of *O. anthropi* or 10^6^ of *B. abortus* (black circles) CFU/mouse. At the indicated times the CFUs per spleen were calculated. (B) HeLa cells treated with CNF for 2 h or non-treated were infected with *O. anthropi* or *B. abortus* at a MOI of 500. Extracellular bacteria were killed with gentamicin (100 µg/ml). At the indicated times intracellular CFU were determined. (C) Raw 264.7 macrophages were infected with the indicated bacteria at a MOI of 500 and processed as in “B”. (D) Lysates of *O. anthropi* (Oa) and *B. abortus* (Ba) were probed with anti-VirB8 and anti-BvrR antibodies. Genomic DNA (1) or cDNA (2) from *O. anthropi* was used as template for PCR amplification of the indicated genes. No amplification from total *O. anthropi* RNA preparation (minus RT) was observed (not shown). All values of *O. anthrophi* in A, B and C were significantly different at p<0.001 with respect to *B. abortus*.

We first compared the replication of *O. anthropi* and *B. abortus* in mice after intraperitoneal injection, a route commonly used in virulence studies [21]. As expected, *B. abortus* reached high numbers one week after infection and maintained those numbers throughout the experiment, reflecting the chronic nature of brucellosis (Fig. 1A). *O. anthropi*, on the other hand, was recovered in four orders of magnitude and CFU numbers diminished throughout the experiment (Fig. 1A). However, under the conditions used, clearance was not complete, indicating some ability to persist. Although at 10^9^ *O. anthropi* bacterial doses not deaths were recorded, piloerection and diarrhea were observed at 48 h after infection, suggesting moderate endotoxicity.

We then studied the ability of *O. anthropi* to invade and multiply in cells. For this, we first infected HeLa cells with *O. anthropi* or *B. abortus* and killed extracellular bacteria with gentamicin. Even at early times, *O. anthropi* was recovered in three orders of magnitude less than *B. abortus*, thus showing its inability to induce internalization (Fig. 1B). This was confirmed by intracellular-extracellular differential fluorescence, which showed that less that 0.1% of HeLa cells interacted with *O. anthropi* and that most of the visualized bacteria were located extracellularly. Moreover, whereas the CFU numbers of intracellular *B. abortus* increased throughout the experiment, those of *O. anthropi* declined steadily (Fig. 1B). In order to bypass the internalization deficiency and to determine if *O. anthropi* was able to multiply intracellularly, we used the *Escherichia coli* Cytotoxic Necrotizing Factor, a toxin that confers a phagocytic phenotype to HeLa cells [22]. This treatment increased the intracellular *O. anthropi* in one order of magnitude (Fig. 1B) and most visualized bacteria were intracellular. Nevertheless, *O. anthropi* was not able to achieve sustained replication, even though some survival was observed (Fig. 1B). Then, we repeated the experiments in Raw 264.7 murine macrophages. Although at early times the intracellular numbers of *O. anthropi* paralleled those of *B. abortus*, the replication curves coursed with opposite tendencies because, whereas *B. abortus* replicated, *O. anthropi* did not (Fig. 1C). Noteworthy, the later bacteria remained cultivable 48 h after infection (Fig. 1C). Altogether, these results indicate that *O. anthropi* possesses a very limited ability to invade and multiply intracellularly in cells.

### *O. anthropi* expresses orthologs of *Brucella* virulence determinants

We have shown that the cell envelopes of *Brucella* play a critical role in virulence [23], [24]. In part, this is due to some unusual physicochemical properties linked to peculiar lipids (i.e. phospholipids, ornithine lipids, LPS lipid A and lipoproteins) and to the properties of the periplasmic cyclic β-glucans. Moreover, the overall outer membrane protein profile [25] and some lipids [26] (Conde-Álvarez et al, unpublished) are under the control of the BvrR/BvrS system, a critical *Brucella* virulence regulator. Also, the envelope is the place where the type IV secretion system VirB is expressed. To contrast these *Brucella* features with those of *O. anthropi*, we first looked for the corresponding orthologs in genomic databases. *O. anthropi* carried orthologs of *Brucella* genes known to be involved in the synthesis of the periplasmic cyclic glucans, ornithine lipids, and phosphatidylcholine. Flagellin genes (coding for a PAMP-bearing protein) were present in *O. anthropi* and *B. abortus*, even though both predicted proteins are modified in the consensus sequence recognized by TLR5 [27]. In addition, *B. abortus* lipoprotein genes *omp19*, *omp16* and *omp10* had orthologs in *O. anthropi*. Other *B. abortus* putative lipoprotein ORFs were also present in *O. anthropi*, although the one encoded by open reading frame (ORF) BAB1_1548 carried a significant deletion encompassing 29 amino acids as compared to its ortholog. However, the converse was not true since orthologs of the putative lipoproteins corresponding to ORFs Oant_3560, Oant_1600, and Oant_2497 were not found in *B. abortus* (Table 1), suggesting a more simple lipoprotein profile in the cell envelope of the latter bacteria. Putative orthologous of genes coding for specialized acyltransferases and acylcarrier proteins (LpxXL and AcpXL) necessary to synthesize and incorporate VLFACs to lipid A were also present.

**Table 1:** Genomic comparison of *Brucella* 2308 and *O. anthropi* LMG3331 ORFs involved in the synthesis of surface exposed or OM PAMPs.

Biosynthetic pathway	ORF			Protein		
	*B. abortus*	*O. anthropi*	Gene	identity-similarity%	Putative protein	Comments (references)
Ornithine lipids	BAB1_0147	Oant_0160	*olsB*	89–93	3-hydroxyacyl-AcpP-dependent acyltransferase	Polar lipids with cationic head that may be counterbalancing negatively charged groups and thus contribute to OM stability
	BAB1_2153	Oant_0759	*olsA*	88–91	1-acyl-sn-glycerol-3-phosphate acyltransferase	
Phosphatidylcholine	BAB2_0668	Oant_3634	*pcs*	82–85	Phosphatidylcholine synthase	Typically eukaryotic phospholipid necessary for full *Brucella* virulence [35], [80]
	BAB1_2131	Oant_0788	*pmtA*	89–94	Phosphatidylethanolamine N-methyltransferase	
Flagellin	BAB2_1106	Oant_4201	*fliC*	55–66	Flagellin	The consensus recognized by TLR5 is modified in both proteins. *O. anthropi* expresses flagellin in vitro. Although expression of flagellin in *Brucella* seems very limited, it plays a role in intracellular survival [81].
LPS core oligosaccharide	BAB1_1522	Oant_1661	*lpcC*	82–89	Glycosyltransferase (family 4)	LPS is a main virulence factor of *Brucella.* Weakly recognized by innate immunity. Although the core, O-chain and lipid A are involved in virulence [20], only the core oligosaccharide and lipid A are part of the LPS PAMP.^2^
	BAB1_0351	Oant_0415	–	58–71	Glycosyltransferase (family 25)	
	BAB1_0639	Absent	wa**		Glycosyltransferase (family 25)	
	BAB1_0535	Oant_0698	*wbkF*	78–87	bactoprenol N-acetylhexosamine-1-phosphate transferase 1	
	BAB1_0534	Oant_0697	*wbkD*	87–93	N-acetylglucosamine 4,6-dehydratase/5-epimerase/3-epimerase 1	
LPS lipid A 3	BAB1_0761	Oant_2558	*lpxE-1*	71–80	LipidA-1-phosphatase (PAP2 family)	This moiety displays low biological activity and endotoxicity [29], [77]
	BAB2_0131	Absent	*lpxE-2*	–	PA-phosphatase related phosphoesterase	
	BAB1_0874	Oant_2371	*acpXL-1*	99–100	Acyl carrier protein	
	BAB1_0484	Oant_0572	*acpXL-2*	100–100	Acyl carrier protein	
	BAB1_0870	Oant_2375	*lpxXL*	92–96	Bacterial lipid A biosynthesis acyltransferase	
	Present 4	Oant_1613	*lpxO*	99–100	lipid A-myristate β-hydroxylase	
Lipoproteins 5	BAB1_1930	Oant_0928	*omp19*	89–94	Omp19	Deletion induces attenuation [82].Weakly recognized by innate immunity [3]
	BAB1_1707	Oant_1220	*omp16*	98–100	Omp16	
	BAB2_0076	Oant_4265	*omp10*	85–92	Omp10	Deletion induces attenuation [82].Weakly recognized by innate immunity [3]
	BAB1_1548	Oant_1635	–	67–73	Lipoprotein	Gap of 29 aminoacids in central section of BAB1_1548
	BAB1_1009	Oant_2075	–	76–85	Rare lipoprotein A	
	BAB1_0858	Oant_2386	–	92–94	Lipoprotein	
	BAB1_0630	Oant_2672	–	78–86	OM lipoprotein-related protein	
	BAB1_2148	Oant_0764	–	82–86	Lipoprotein	
	BAB1_1041	Oant_2110	–	78–87	Lipoprotein	
	BAB1_0758	Oant_2564	–	86–95	Lipoprotein	
	Absent	Oant_3560	–	–	OM lipoprotein	
	Absent	Oant_1600	–	–	Lipoprotein	
	Absent	Oant_2497	–	–	5′-nucleotidase, lipoprotein E(P4) family	
	BAB1_2147	Oant_0765	–	72–79	Lipoprotein	
	BAB1_1441	Oant_1752	–	93–97	Lipoprotein	
	BAB1_0907	Oant_2339	–	81–87	NlpD-like protein	Identified in proteomic analysis of *B. abortus* [25]
	BAB1_1227	Oant_1984	–	87–92	Omp22K	
	BAB1_1226	Oant_1985	–	90–95	OmpA family protein	
Cyclic β 1–2 glucan	BAB1_0108	Oant_0124	Cgs	92–94	cyclic β 1–2 glucan synthetase	Cyclic β 1–2 glucans are involved in *Brucella* virulence and intracellular trafficking [34]
	BAB1_1017	Oant_2082	–	93–97	cyclic β 1–2 glucan ABC transporter	
	BAB1_0629	Oant_2673	FeuQ	87–93	Sensor protein kinase	
	BAB1_0628	Oant_2674	FeuP	92–96	Regulatory protein	

1 Although acting in the O-PS biosynthetic pathway, these proteins are involved in the steps previous to O-sugar polymerization and, therefore, the corresponding sugars belong structurally to the core oligosaccharide.

2 These bacteria differ in O-PS sugar structure and have unrelated glycosyltransferases and O-PS export systems. Accordingly, only ORFs assigned to the core and lipid A are presented in the Table. Proteins known to be involved in ancillary pathways of *Brucella* core oligosaccharide (Pgm, ManBcore and ManCcore) are highly conserved in *O. anthropi*.

3 Only ORFs attributed in the annotations in databases to the lipid A modification systems [83] are included.

4 This ORF, although not annoted in the *B.abortus* 2308 genome, was identified by using Oant_1613 as a query in the TBLASTN program (NCBI).

5 For lipoproteins, lipoprotein annotations were obtained from both genomes and verified with the lipoP (http://www.cbs.dtu.dk/services/LipoP/) in those cases in which there were discrepancies between the two annotations.

We then examined the presence and expression of the two component sensory-regulatory system BvrR/BvrS in *O. anthropi*. The predicted proteins of the consecutive ORFs Oant_0827 and Oant_0828 show 90.5% and 97.5% identity with BAB1_2092 (*B. abortus* BvrR) and BAB1_2093 (*B. abortus* BvrS), respectively. Primers designed on these sequences gave positive amplification reactions from both *O. anthropi* genomic DNA and cDNA, thus demonstrating transcription (Fig. 1D). In addition, an antibody against *B. abortus* BvrR readily detected a single band in *O. anthropi* lysates with a molecular weight consistent with that of the predicted protein (Fig. 1D).

Finally, we searched for orthologs of the type IV secretion system VirB. *O. anthropi* genome contains two DNA regions with an organization and identity compatible with the presence of a *virB* operon. The first one contains twelve consecutive ORFs (from Oant_674 to Oant_685) with an organization similar to that of the *virB* operon in *B. abortus*. With the exception of Oant_0679, which encodes a hypothetical protein of unknown function, all other ORFs have orthologs in the *B. abortus virB* operon, with percentage identities between the homologous predicted proteins ranging from 22.9% to 45.5%. In contrast, the second DNA region contains only seven ORFs (Oant_4564 to Oant_4562, Oant_4559 to Oant_4557 and Oant_4555) and the predicted proteins show a lower identity (from 18.1% to 35.1%) with the corresponding *B. abortus* VirB products. Primers designed on the sequences of the first DNA region for the *virB4* (Oant_0677), *virB5* (Oant_0678) and *virB8* (Oant_0682) orthologs gave a positive amplification from *O. anthropi* genomic DNA and cDNA indicating their expression (Fig. 1D). Moreover, while the BvrR orthologous protein strongly reacted in Western blots, antibodies specific for *B. abortus* VirB8 showed only a weak band of the appropriate molecular weight in *O. anthropi* lysates (Fig. 1D). These results demonstrate the closer proximity between the BvrR/BvrS orthologs and indicate that in the *Brucellaceae* there is higher degree of divergence in the type IV secretion VirB systems committed to association with eukaryotic cells than in the BvrR/BvrS systems devoted to cell envelope homeostasis.

### *O. anthropi* triggers leukocyte recruitment that lay between those induced by *B. abortus* and *S. enterica*

**Figure 2:**
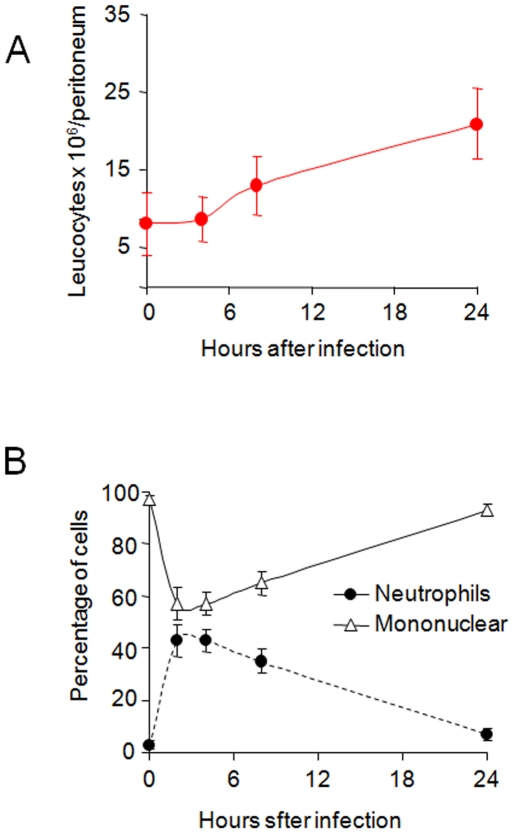
*O. anthropi* recruits leukocytes in the peritoneum. Groups of 6 mice were intraperitoneally inoculated with 10^6^ CFU/ml of *O. anthropi*. Thereafter, peritoneal exudates were obtained and leukocytes counted. (A) Total number of peritoneum leukocytes (B) Relative number of mononuclear (monocytes and lymphocytes) and polymorphonuclear neutrophil leukocytes.

The initial phases of *B. abortus* infection are characterized by a negligible activation of proinflammatory mechanisms which contrasts with those triggered by most Gram negative pathogens [3]. We first tested to what extent this property was shared with *O. anthropi*. As shown in Fig. 2A, *O*. *anthropi* induced recruitment of leukocytes into the peritoneal cavity of mice, with more than a twofold increase in 24 hours. The analysis of these leukocytes indicated a rapid switch, with neutrophils increasing from 3% to more than 40% at two hours after intraperitoneal inoculation (Fig. 2B).

**Figure 3:**
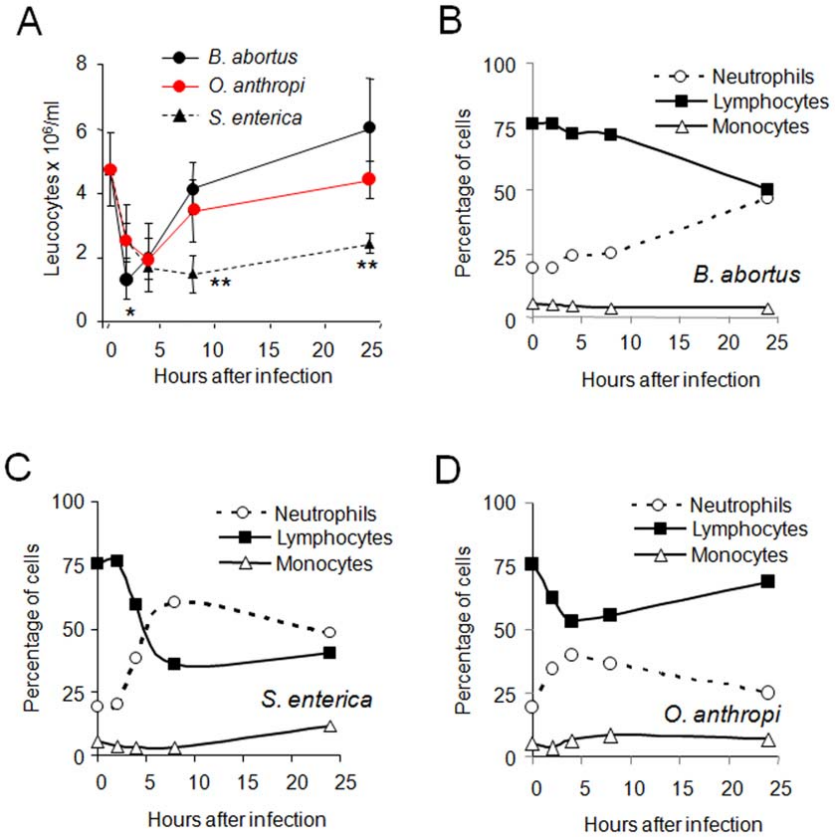
*O. anthropi* infection induces an intermediate blood leukocyte response. Groups of 6 mice were inoculated intraperitoneally with10^6^ CFU/ml of the indicated bacteria, bleed at different times and leukocytes counted in blood. (A) Total leukocytes number of mice infected with the indicated bacteria. (B) Relative blood leukocyte number of mice infected with *B. abortus*. (C) Relative blood leukocyte number of mice infected with *S. enterica*. (D) Relative blood leukocyte number of mice infected with *O. anthropi.* Values of *O. anthropi* in “A” were significantly different at p<0.01 (*) and p<0.001 (**) with respect to *B. abortus.* In “B, C and D” the standard error was less that 5% at all points.

We also monitored the changes in blood after peritoneal inoculation in comparison with *B. abortus* and *S. enterica*. Although there was a quick initial decrease in the circulating leukocytes in the three cases, numbers remained low in mice inoculated with *S. enterica*. In contrast, there was a rapid return to basal levels in *B. abortus* inoculated mice and an intermediate phenotype, slightly closer to that of *B. abortus*, obtained with *O. anthropi* (Fig. 3A). The evaluation of the blood leukocyte populations showed a moderate change in the proportion of neutrophils in mice inoculated with *B. abortus* (Fig. 3B) and a rapid increase of the same cells in mice inoculated with *S. enterica* (Fig. 3C). Again, *O. anthropi* triggered an intermediate response (Fig. 3D).

### Neutrophils control *O. anthropi* but not *B. abortus* infections

**Figure 4:**
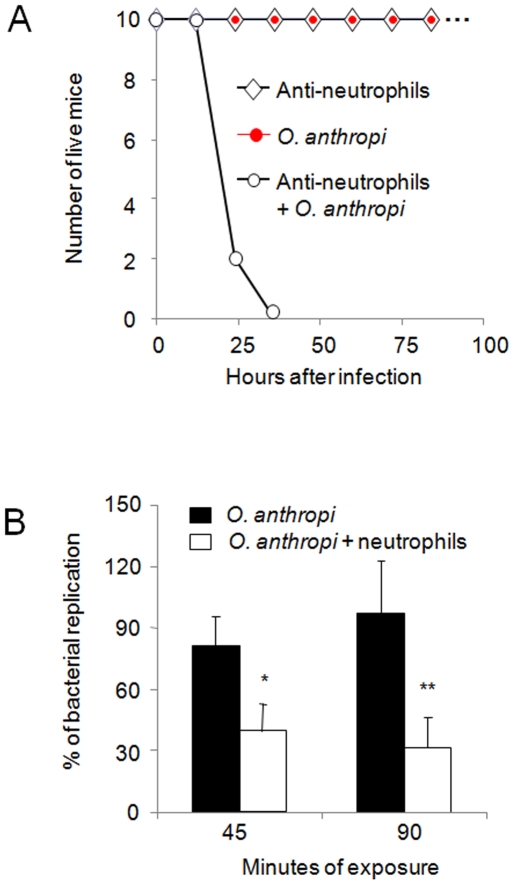
Neutrophils are required for the control of *O. anthropi* infections. (A) Neutrophils were depleted from twenty mice by injection of the anti-neutrophil RB6 antibody and ten additional mice were instead injected with PBS alone. One group of ten mice anti-neutrophil treated and the group of ten mice injected with PBS alone were then infected intraperitoneally with 10^9^ CFU/mouse of *O. anthropi*. The last group of ten mice treated with antibody anti-neutrophil was inoculated with PBS alone. The lethality of the bacteria was recorded at the indicated times. (B) *O. anthropi* was mixed with purified rat neutrophils at a ratio of 5±4 bacteria/cell. Control bacteria were not incubated with neutrophils. At the indicated times the viable CFU were determined and the percentage of bacterial replication was calculated. In “B” Values of p<0.01 (*) and p<0.001 (**) are indicated.

The above results indicated that *O. anthropi* induces a cellular response compatible with acute bacterial infections and suggested that neutrophils could play a protective role. In addition, we previously observed that neutrophils do not play a significant role in *B. abortus* infections in mice [3]. Thus, we evaluated the relevance of these leukocytes in controlling *O. anthropi* using chronic neutropenic mice. Forty-eight hours after infection, all the neutropenic but none of the non-neutropenic mice were dead (Fig. 4A). The role of neutrophils in controlling *O. anthropi* was confirmed by the results of *ex-vivo* experiments with rat neutrophils (Fig. 4B).

### *O. anthropi* activates complement through non-LPS determinants

**Figure 5:**
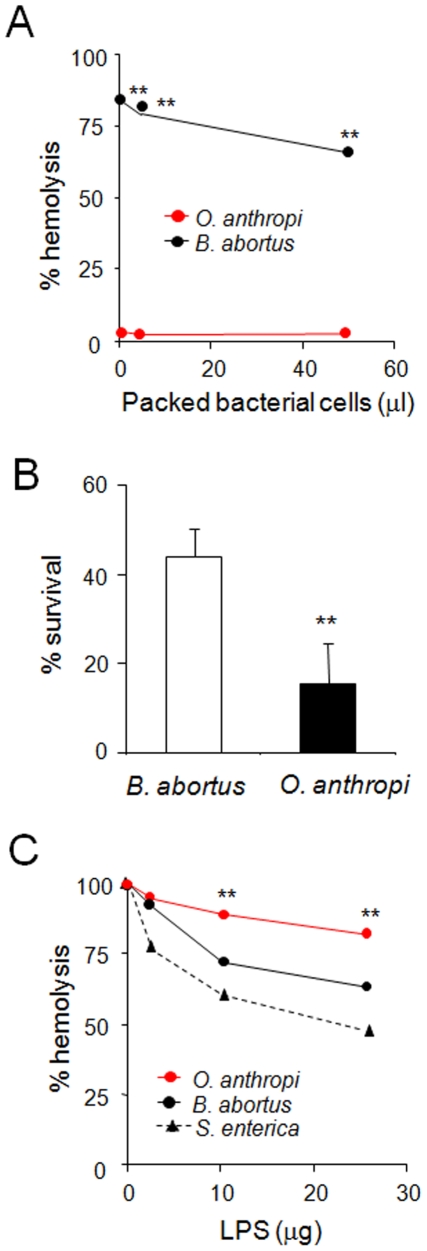
*O. anthropi* cells but not its LPS fix complement. (A) Rabbit serum was incubated with the indicated amounts of *O. anthropi* or *B. abortus* cells. After incubation, the ability of the serum to lyze pre-sensitized erythrocytes was determined and expressed as percentage of hemolysis. (B) The indicated bacteria were incubated in the presence of bovine serum for 90 min and their relative resistance was calculated.(C) Rabbit serum was incubated with the indicated concentrations of *Oa*LPS, *Ba*LPS or *Se*LPS. After incubation the ability of the serum to lyse pre sensitized erythrocytes was determined. Values of *O. anthropi* in were significant different at p<0.001 (**) with respect to *B. abortus*.

Both *Brucella* cells and LPS are very poor complement activators [3]. To assess whether *O. anthropi* shares this property, we incubated rabbit serum with *B. abortus* and *O. anthropi* and then measured the lytic activity remaining in the supernatants using antibody-sensitized erythrocytes. Whereas *B. abortus* practically did not consume complement, *O. anthropi* completely abrogated the lytic activity, demonstrating an effective surface binding and the subsequent activation (Fig. 5A). Next, we tested the bactericidal activity of bovine serum against these bacteria. In agreement with the high avidity of the complement for the *O. anthropi* surface, this bacterium was considerable more susceptible to the bactericidal action of serum than *B. abortus* (Fig. 5B). Since LPS is one of the main molecules responsible for complement activation by Gram negative cell surfaces, we tested whether the *O. anthropi* LPS (*Oa*LPS) consumed complement. Surprisingly, *Oa*LPS was less effective than *Ba*LPS in depleting complement activity, even both were less effective than the LPS from *S. enterica* (*Se*LPS) (Fig. 5C). Therefore, other *O. anthropi* surface molecules, such as capsule polysaccharides, may be responsible for binding complement.

### *O. anthropi* and its LPS trigger cytokine responses that lay between those of *B. abortus* and *S. enterica*

**Figure 6:**
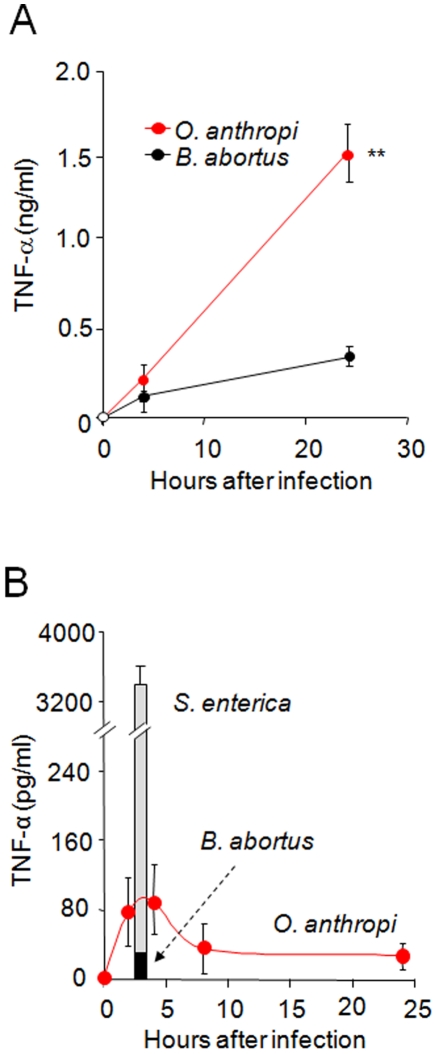
*O. anthropi* induces TNF-α that lays between *B. abortus* and *S. enterica*. (A) Murine Raw 264.7 macrophages were infected for 30 min with *O. anthropi* or *B. abortus* at a MOI of 500. Extracellular bacteria were killed by addition of gentamicin. At the indicated times samples from the culture media were taken and processed for TNF-α quantification. (B) Groups of 6 mice were inoculated intraperitoneally with *O. anthropi*. At the indicated times mice were bled and TNF-α levels were determined. The levels of TNF-α induction after 3 h of an equivalent inoculation of *B. abortus* or *S. enterica*. Value of p<0.001 (**) is indicated.

Proinflammatory cytokines, particularly TNF-α, increase during acute Gram negative infections but not at the onset of brucellosis. When we studied this in comparative terms, we found that murine macrophages infected with *O. anthropi* released significantly higher amounts of TNF-α than cells infected with *B. abortus* (Fig. 6A), and this observation held true *in vivo* since mice displayed higher TNF-α blood levels when inoculated with *O. anthropi* than with *B. abortus* (Fig. 6B). *O. anthropi*, however, was not as efficient as *S. enterica* in the induction of TNF-α release *in vivo* (Fig. 6B).

**Figure 7:**
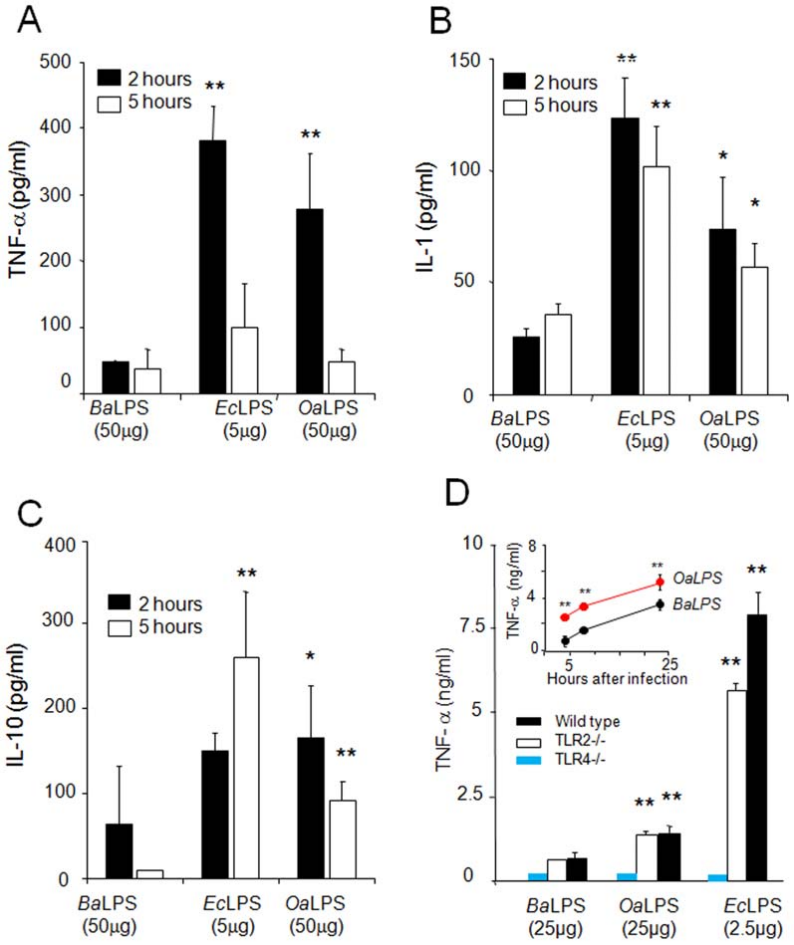
*Oa*LPS induces cytokine responses that lay between *Ba*LPS. and *Se*LPS. (A, B and C) Groups of 6 mice were inoculated intraperitoneally with *Ba*LPS, *Ec*LPS or *Oa*LPS at the indicated concentrations. Mice were bled at 2 or 5 h and interleukins quantitated by ELISA. (D) Bone marrow macrophages from wild type TLR2−/− and TLR4−/− knockout mice were incubated with the indicated LPSs. After incubation TNF-α was quantified in the supernatant of cells at 24 h. The insert shows Raw 264.7 macrophages incubated with 25 µg of *Oa*LPS or *Ba*LPS and at the indicated times TNF-α quantified from cell culture supernatants. Values of p<0.05 (*) and p<0.005 (**) with respect to *Ba*LPS are indicated.

Since LPS is the foremost PAMP-bearing molecule of Gram negative bacteria, we compared the ability of *Oa*LPS, *Ba*LPS, and *E. coli* LPS (*Ec*LPS) to trigger cytokine responses in mice. *Oa*LPS consistently induced higher blood levels of TNF-α, IL-1 and IL-10 than *Ba*LPS (Fig. 7A–C) but lower than those induced by *Ec*LPS, even if the latter was used at ten times less concentration (Fig. 7A–C). Consistent with these results, *Oa*LPS induced a higher cytokine release in cultured macrophages than *Ba*LPS (Fig. 7D insert). The induction of TNF-α release by *Oa*LPS was TLR4- but not TLR2-dependent, because only macrophages derived from TLR4-knockout mice failed to release this cytokine (Fig. 7D). This pattern was similar to the induction of TNF-α by *Ec*LPS, which also depended on TLR4 and not on TLR2 (Fig. 7D). Under the conditions used, the TNF-α levels produced by macrophages stimulated with *Ba*LPS were significantly lower in both TLR2- and TLR4-deficient cells (Fig. 7D).

### *Oa*LPS partially induces dendritic cell maturation

**Figure 8:**
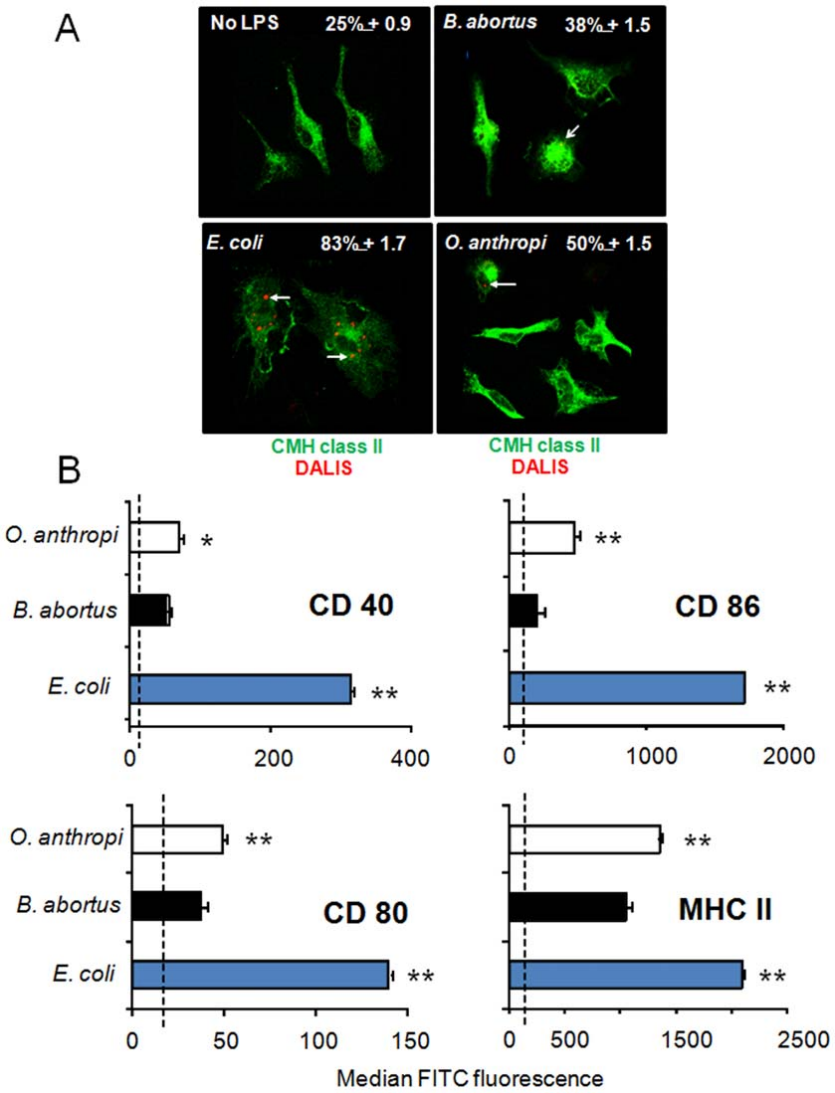
*Oa*LPS induces dendritic cell maturation that lay between *Ba*LPS. and *Se*LPS. (A) BMDC were incubated with 0.1 µg of *Ec*LPS, 10 µg of *Ba*LPS or 10 µg of *Oa*LPS in 1 ml culture and labeled with the anti-FK2 antibody for detection of DALIS at 24 h. The percentage of BMDC positive for DALIS is indicated. (B) BMDC incubated as in “A” were labeled with fluorescent anti-CD40, CD-80, CD-86 and MHC II. Values of median fluorescence correspond to four independent experiments. Values of p< 0.05 (*) and p<0.005 (**) with respect to *Ba*LPS are indicated.

Dendritic cells are key elements linking innate and adaptive immunity. Since the evidence presented above situates the induction of innate immunity by *O. anthropi* between those elicited by enteric bacteria and *B. abortus*, we wanted to know whether this was also true in these cells. To this purpose, we measured the ability of the respective LPSs to trigger the formation of the dendritic cell aggregosome-like induced structures (DALIS) characteristic of the maturation of these cells. As expected, *Ec*LPS induced more DALIS than *Ba*LPS. Moreover, in keeping with all the above-described results, *Oa*LPS displayed an activity that lay between *Ba*LPS and *Ec*LPS (Fig. 8A). To confirm this, we measured the expression of maturation markers in LPS-treated dendritic cells. As compared to *Ba*LPS, *Oa*LPS induced a significantly higher expression of surface CD40, CD80, CD86 and MHC-II which was, however, lower than that triggered by *Ec*LPS (Fig. 8B).

### *Oa*LPS partially activates NF-κB and its affinity for MD-2 lies between that of *Ba*LPS and *Se*LPS

**Figure 9:**
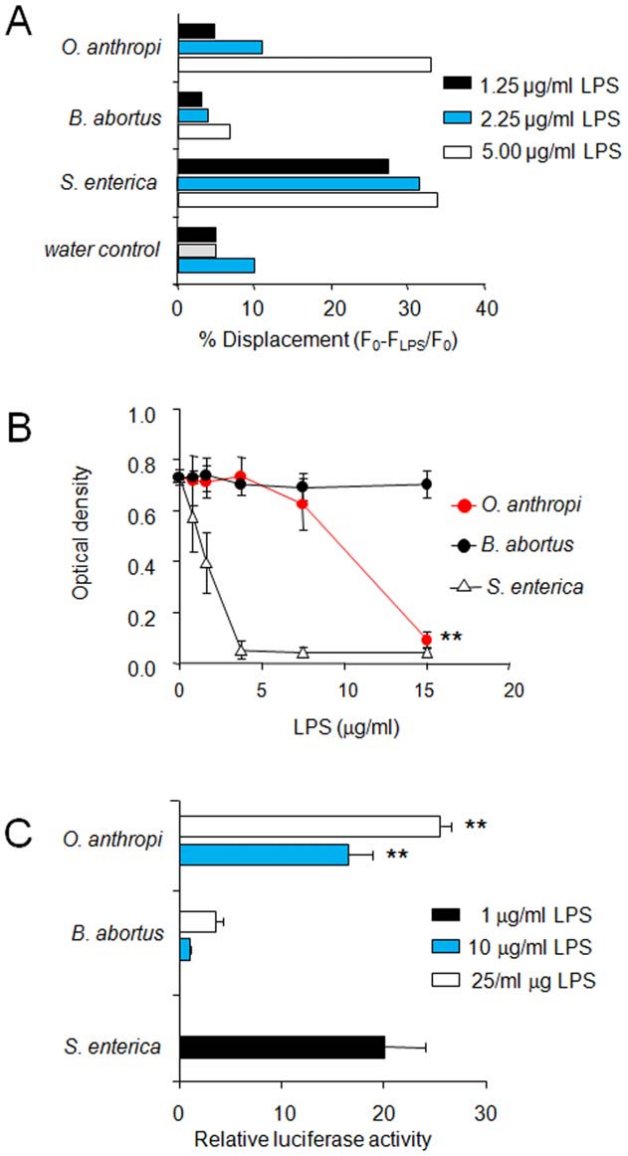
*Oa*LPS shows an intermediate affinity for MD-2 and partially activates NF-kB. (A) Displacement of bis-ANS by LPS from the bis-ANS/hMD2 complex. The bis-ANS/hMD2 complex (50 nM/50 nM) was incubated for 5 minutes to reach stable fluorescence (F0). Then, increasing amounts of the indicated LPS or the same volume of water were added and fluorescence displacement measured. (B) Detection of free hMD2 by ELISA after incubation (0.75 mM of hMD2) with increasing concentrations of the indicated LPS. (C) NF-κB expression measured by relative luciferase activity of HEK293 transfected cells with hTLR4, hMD2, human CD14, NFκB-luc and Renilla containing plasmids upon respective LPS stimulation. The responsiveness to LPS was determined by the NF-κB-responsive luciferase reporter plasmid, which was normalized using constitutively active Renilla luciferase. Value of p<0.005 (**) with respect to *Ba*LPS is indicated. In *Se*LPS all values beyond 0.1 µg were significant at p<0.005, with respect to *Ba*LPS.

Most biological effects of LPS depend on its interaction with the TLR4 co-receptor MD-2, an event that triggers a cascade of signals leading to the NF-κB-dependent activation of immune response genes. On the basis of the above-described results, we hypothesized that *Oa*LPS would have a higher binding to MD-2 than *Ba*LPS but lower than *Se*LPS, and that this would lead to an NF-κB activation between both types of LPSs. To test this possibility, we compared first the ability of *Oa*LPS, *Ba*LPS, and *Se*LPS to displace the hydrophobic probe bis-ANS from the binding site of human MD-2 (hMD2) [28]. We found that *Se*LPS was able to displace approximately 30% of bis-ANS from the hMD-2-binding site at concentrations from 1.25–5 µg/ml (Fig. 9A). Displacement by *Ba*LPS, on the other hand, remained close to the background values obtained with an equal amount of water (Fig. 9A). At the highest concentration tested, *Oa*LPS caused a displacement similar to that induced by *Se*LPS. However, at lower concentrations it approached the negligible values of *Ba*LPS (Fig. 9A). The interaction with hMD-2 was also measured as the ability of the LPSs to block the recognition of hMD2 by anti-hMD-2 monoclonal antibody (Fig. 9B). *Se*LPS inhibited this antibody in a dose-dependent manner, with a 50% reduction in the signal at a concentration of 2 µg/ml. *Ba*LPS did not reduce significantly the binding of the anti-hMD-2 antibody at any of the concentrations tested. Again, *Oa*LPS showed an activity that laid between *Ba*LPS and *Se*LPS since it blocked the antibody at concentrations higher than those of *Se*LPS (Fig. 9B). Finally, we assessed the activation of the terminal end of the signaling pathway by measuring the relative activity of a luciferase reporter under the control of the NF-κB promoter in HEK 293 cells transfected with expression vectors containing hMD-2, human TLR4 (hTLR4) and human CD14 (hCD14). As can be seen in Fig. 9C, 10 to 25 times higher concentrations of *Oa*LPS were necessary to generate a relative luciferase activity similar to that induced by 1 µg/ml of *Se*LPS. Consistent with other observations, *Ba*LPS induced a weaker luciferase activity (Fig. 9C).

## Discussion

We proposed that *B. abortus* follows a stealthy strategy which relies on the absence, modification and inaccessibility of PAMP-bearing molecules behind a firm outer membrane [3]. A related hypothesis is that this pathogen has evolved from a free living α-*Proteobacteria* bacterium with envelope structural features already representing a first step towards innate immunity evasion [3], [18], [29]. According to this, the *Brucella* ancestor underwent further changes in such a structural scaffold upon contact with innate immunity effectors and, in parallel, reduced its genome, removed plasmids and trimmed-down its metabolic alternatives [30]. The results presented here reinforce these hypotheses in three ways: i), they show that the proinflammatory response to *Brucella* is significantly lower than that to the closest known neighbor bacteria which, significantly, is only an opportunistic pathogen; ii) they demonstrate the existence of envelope molecules prone to low recognition in *Brucellaceae* members that are primarily soil inhabitants and; iii) they support the notion that relatively few and not drastic structural changes in a key PAMP-bearing molecule result in very different lifestyles.

The early clinical evolution of human brucellosis is characterized by a negligible pro-inflammatory response [31], a phenomenon reproduced in experimental models [3]. In contrast, *O. anthropi* infection triggered leukocytosis, neutrophilia, platelet aggregation and acute fever [32], [33], all indicative of sepsis. The relevance of this response in *O. anthropi* infections is best exemplified by the prominent role played by neutrophils. These cells were bactericidal for *O. anthropi* and their absence converted an otherwise harmless microbe into a lethal pathogen. This agrees with the opportunistic character of *O. anthropi* and sets a clear cut limit with brucellosis, where neutrophils do not seem to play a significant role in controlling the infection [3]. It is tempting to speculate that conserved molecules involved in *Brucella* virulence like phosphatidylcholine, the VLCFA in lipoproteins and lipid A, the periplasmic cyclic glucans, and the orthologous BvrR/BvrS and VirB systems [34], [35], [36] participate in this survival. In the natural environment of *Ochrobactrum*, these structures and systems may be related to resistance to antibiotic peptides, osmotic stress, the transference of plasmids, survival against protozoan predation, or other modes of interaction with eukaryotic cells of the rhizosphere [4], [37].

Similarly to the proinflammatory response, It is striking that the adaptive immune response provoked by *O. anthropi* also lies between that induced by typical extracellular and intracellular pathogens [38]. Indeed, while *Brucella* infection is characterized by a protective Th1 immune response [39], *O. anthropi* infection stimulates a mix of Th1 and Th2 type response which does not protect against *Brucella* [38]. However, when switched to a Th1-biased response by addition of the TLR9 agonist unmethylated CpG, *O. anthropi* induces some protection against *Brucella*. Since protection against *Brucella* is dependent on TLR9 and Myd88 [40], [41] and, since unmethylated CpG is not accessible unless envelopes are breached, then the toughness of the *Brucella* envelope should play an important role in the stealthy behavior. The results presented here and previously [18] evidences this and demonstrates that *O. anthropi* differs from *Brucella* in crucial envelope properties.

It is feasible to propose that *Brucella* ancestors had already envelope molecules displaying determinants that departure from the PAMPs targeted by innate immunity receptors. Since PAMPs are conserved because they are functionally essential, one may ask why this departure from the canonical structure was possible in the *Brucellaceae*, and what kind of selective forces were behind it. In most *Proteobacteria*, cell envelopes are impermeable to a large variety of noxious substances through the combined action of outer leaflet characteristics that do not allow the partition of hydrophobic substances, a property linked to the high density of negatively charged groups in the outer membrane [42], [43]. This leads to the conservation of those groups and, consequently, to their ready recognition as PAMPs by innate immunity, as illustrated by the wide array of bactericidal peptides that exert their action upon binding to the negative charged groups in core and lipid A.

Whereas *O. anthropi* keeps the permeability barrier, *Brucella* does not and is concomitantly less sensitive to bactericidal peptides [18]. Significantly, this property also lays between the high resistance of *Brucella* envelopes and the sensitivity of enterobacterial envelopes to bactericidal cationic peptides [18]. This shows that *Brucella* and *O. anthropi* represent a lineage endowed with outer membranes and LPSs with some degree of resistance to bactericidal peptides and, since the primary habitat of most *Brucellaceae* is the soil, it is likely that this property resulted from the selective pressure of antibiotic peptides produced by soil microorganisms. However, in *Brucella* the permeability barrier to which sensitivity to bactericidal peptides is linked, is no longer necessary to confront mucosal surfaces or intracellular environments. Therefore, this property could have been relinquished by the *Brucella* ancestor, keeping and increasing its resistance to host bactericidal peptides.

**Figure 10:**
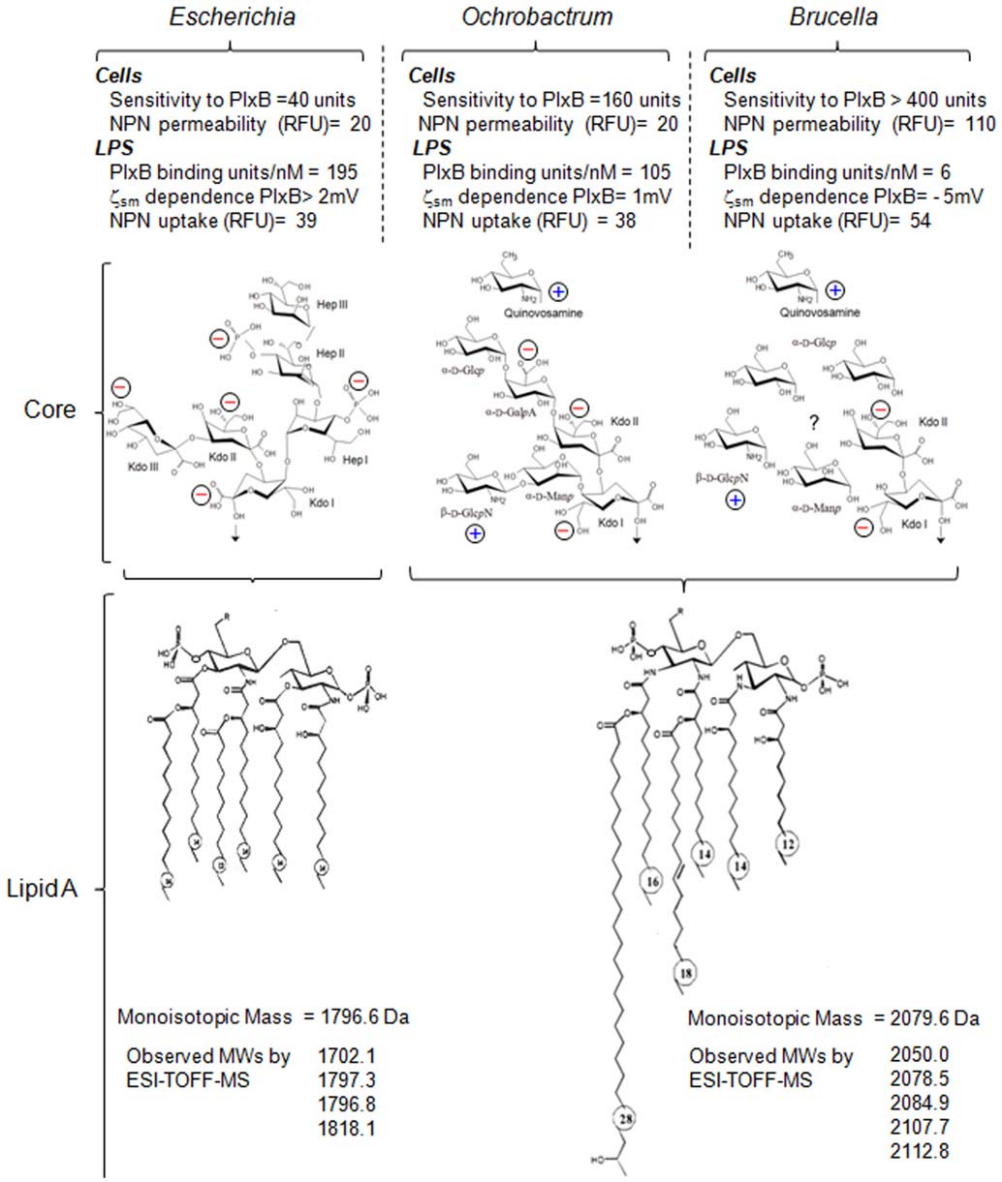
Model of *Brucella, Ochrobactrum* and *Escherichia* lipid As and core oligosaccharide moieties. [18], [19], [45]. The presence of three KDO and phosphorylated heptoses confers five negative charges to the *Escherichia coli* core oligosaccharide. The core oligosaccharide of *Ochrobactrum* LPS possesses galacturonic acid, in contrast to *Brucella* LPS core which does not possess this negatively charged acidic sugar. The differential sensitivity of *Escherichia*, *Ochrobactrum* and *Brucella* to cationic peptides expressed as sensitivity to polymyxin B (PlxB) units (U) is linked to the different LPSs properties such as uptake hydrophobic probes (NPN), PlxB binding, and LPS zeta potential (ζ) dependence after saturation with polymyxin B [18]. Notice that upon saturation with PlxB, zeta potential became positive (1 mV) for *Ochrobactrum* LPS while remaining negative (−5 mV) for *Brucella* smooth LPS, suggesting hindered access to inner targets. *Ochrobactrum* and *Brucella* LPSs does not show significant differences in lipid A structure and acyl chain fluidity and display very similar structure [18], [19], [45]. It has been proposed that the quinovosamine sugar in *Ochrobactrum* and *Brucella* LPS links the O core oligosaccharide with the O-chain polysaccharide [21].

An analysis of the respective LPSs, supports above developed hypotheses (Fig. 10). The similarity of the biological activity of the *Ba*LPS and *Oa*LPS makes unlikely that the respective O-chains play a significant role since they are very different structures [44], [45] and, at least for *B. abortus* there is evidence for horizontal acquisition [46]. On the other hand, the VLCFAs of both bacteria represent a first departure of the canonical lipid A structure (Fig. 10) that seems ancestral in α-2 *Proteobacteria* [47]. Since VLCFAs carry an hydroxyl group at the iso position and are postulated to span the outer membrane [47], they could provide a firm anchorage that contributes to explain the integrity of the outer membranes observed in the polymyxin B binding experiments [18]. Detail core structures have not been elucidated for any *Brucella* species or *O. anthropi*, but the available information on the *Brucella* closer relative *O. intermedium* and on the sugar and lipid compositions in *B. abortus* and *O. anthropi* also shows a departure from enteric bacteria, with a comparatively lower number of negatively charged groups (Fig. 10). Moreover, quantitative analysis suggests a further charge reduction in *Brucella* LPS core that is consistent with the loss of the permeability barrier and its greater resistance to bactericidal peptides (Fig. 10).

The departure from the canonical PAMP in both the VLCFAs of lipid A and in the negatively charged groups of the lipid A-core of *Ochrobactrum*, enhanced further in *B. abortus*, is in agreement with the results of the LPS-hMD-2 binding experiments. MD-2 comprises a hydrophobic pocket and some cationic domains that correspond to two functional types of LPS recognition sites, one for the lipid A portion and the other for the anionic groups [28], [48], [49]. In the case of the lipid A, it would be impossible to accommodate all acyl chains, particularly VLCFA, into the hydrophobic pocket of MD-2. A model of TLR4 activation, that suggests that the secondary acyl chain of the lipid A protrudes out of the MD-2 pocket and directly interacts with hydrophobic side chains of TLR4 (F440 and F463), diving the receptor dimerization and activation, has been suggested [50]. In case of *Oa*LPS we propose that the VLCFA interact directly with TLR4, and in this case the terminal hydroxyl group increases its propensity to be exposed to the solvent. The key requirement for activation of TLR4.MD-2 complex is the delivery of monomeric LPS, by LBP and CD14, which may also be determined by the LPS core, which differs between the *Ochrobactrum* and *Brucella* LPSs (Fig. 10). Since these LPS structures are rooted in the phylogeny of the group, this illustrates how relatively few structural changes on an orthologous structure may contribute to adaptation to widely different lifestyles.

Not all properties of *O. anthropi* were related to LPS. The complete bacterium readily consumed complement and was killed by serum, but its LPS did not trigger the lytic cascade. Consequently, differences in other surface structures, such as *O. anthropi* lipoproteins or extracellular polysaccharides [51], should be responsible for complement activation and the ensuing proinflammatory activities. Bacterial capsular polysaccharides have been shown to consume complement and stimulate cytokine production through TLR activation [52], [53], [54].

Animal pathogenic α-*Proteobacteria* have molecules not readily recognized by innate immunity, and these bacteria also tend to establish chronic infections [55], [56], [57], [58], [59]. For instance, *Bartonella* possesses a low agonist non-canonical lipid A with VLCFA, a low number of lipoproteins and flagella not recognized by TLR5 [27], [59], [60]. In *Rickettsia*, there is an absence of flagella a reduced number of lipoproteins and a non-canonical LPS [61], [62]. The insect *Wolbachia* parasites do not posses LPS, flagellum, fimbria or pili, and contain a low number of lipoproteins and an unusual peptidoglycan [63]. Intracellular *Ehrlichia* and *Anaplasma* have low number of lipoproteins and do not have LPS, peptidoglycan or flagellin [64], [65]. Therefore it seems plausible that the α-*Proteobacteria* intracellular pathogen ancestor may have been endowed with structures prone to low recognition. In addition, animals could have developed an innate immune repertoire that preferably recognizes β and γ-*Proteobacteria* rather than α-*Proteobacteria* molecular patterns. In the case of *Brucella*, it is possible that evolutionary pressures linked to innate immunity forced for the selection of organisms displaying low PAMPs recognition [66]. The selective forces working in the second scenario could be linked to the early endosymbiotic relationship between eukaryotic cells and α-*Proteobacteria* ancestors [67] which would have led to the identification of some bacterial molecules as “self” in relation to those of β and γ-*Proteobacteria*, which appeared later in evolution [68]. It is worth noting, however, that all these α-*Proteobacteria* have orthologous type IV secretion systems, which seem to be essential for controlling the intracellular survival.

We propose that, *Brucella* evolved from an opportunistic pathogen, non markedly endotoxic ancestor within a mammal environment [30], [69] by: i), removing microbial molecular determinants such as capsules and fimbriae; ii), modifying surface molecules such as flagella, lipoproteins and LPS to provide high resistance to bactericidal cationic substances and complement and, concomitantly, avoid recognition by TLRs; iii), keeping structures such as peptidoglycan and unmethylated DNA under a tough envelope; and iv), maintaining the regulatory systems (e.g. BvrR-BvrS) that control the homeostasis of an outer membrane whose overall structure is essential in the interaction with the host. This furtive strategy, which also required a remodeling of ancestral type IV secretion systems to sneak and reproduce in cells, does not preclude the development of determinants actively hampering the activation of immunity at later stages [70], [71], [72].

Finally, the recorded phenotypic, biological and pathogenic differences between *Brucella* and *Ochrobactrum*, strengthens arguments for maintaining these two bacterial clusters as separate genera in the *Brucellaceae* and reinforce the concept that non-drastic molecular variations generate marked divergences that result in different biological groups.

## Materials and Methods

### Ethics Statement

All animals were handled and sacrificed according to the approval and guidelines established by the “Comité Institucional para el Cuido y Uso de los Animales of the Universidad de Costa Rica”, and in agreement with the corresponding law “Ley de Bienestar de los Animales N^o^ 7451” of Costa Rica (http://www.protecnet. go.cr/salud). The recommendations stated in the Weatherall report “The use of non-human primates in research” (http://www.mrc.ac.uk/Utilities/Documentrecord/index.htm?d=MRC003440), were followed accordingly.

### Bacterial strains, LPSs and biological reagents

*Salmonella enterica* sv. *typhimurium* strain SL1344, virulent *B. abortus* 2308, and *O. anthropi* LMG 3331^T^ have been previously described [1], [45], [73]. *Ba*LPS, *Oa*LPS, *Se*LPS and *Ec*LPS were purified and characterized as previously described [18], [24]. *S. enterica* (strainHL83) LPS was provided by K. Brandenburg (Forschungszentrum Borstel, Germany). *E. coli* CNF was purified as previously described [22]. BvrR was cloned as glutathione-S-transferase (GST) fusion protein in pGEX-2TK vector, expressed in *E. coli* BL21(DE3), and purified following standard protocols. Rabbit anti-BvrR antibodies were produced by four intramuscularly applied boosts of GST-BvrR (250 µg) in complete (first boost) or incomplete (second to fourth boost) Freund adjuvant (Sigma-Aldrich Co.). Antibodies from 2 ml of serum were adsorbed to GST-BvrR-Sepharose beads, eluted with 0.2 M glycine, pH 2.5, and collected in 1 M Tris, pH 9,0. The antibodies were concentrated by ultrafiltration and stored at -20°C in 50% glycerol. The anti-*Ochrobactrum* antibody was produced in a rabbit by 4 boosts of intramuscular injections of formaldehyde fixed bacteria. The specificity of the antibody was confirmed by immunofluorescence against a panel of related α-*Proteobacteria*. Enzyme linked immunosorbent assay (ELISA) kits for cytokine determination were from BD Biosciences (San Diego, CA.). Monoclonal antibody RB6-8C5 against murine granulocytes was a gift from Bärbel Raupach, MPIIB, Berlin, Germany. Allophycocyanin conjugated-anti-CD11c antibody (HL3) and phycoerythrin-conjugated anti-CD40, -CD80, -CD86, and -MHC class II were all from Pharmingen. Mouse antibody FK2 (Biomol) was provided by D. Williams (University of Toronto). The properties and reactivity of anti-VirB8 antibody have been described before [74].

### Infections and bacterial counts in experimental animals

The characteristics, source and maintenance of Balb/c, C57BL/6 and the KO counterparts TLR4−/− and TLR2−/−, mice have been described previously [3], [72]. Generation of neutropenic mice was performed as described before [3]. For calculating the bacterial multiplication rates in spleens, groups of 20 eight-week-old female BALB/c were intraperitoneally inoculated with 0.1 ml of a suspension of the indicated bacteria species. Animals for each group were killed by cervical dislocation at appropriate times. Spleens were aseptically removed and individually homogenized in 1 ml PBS. Dilutions from this homogenate were seeded on TSA and CFU numbers determined. Alternatively mice were inoculated with 0.1 ml (pyrogen-free sterile PBS) of the indicated LPS preparation by the intraperitoneal route. Wistar rats were maintained in the animal facility of the Veterinary School of the National University, Costa Rica. Cytokine or leukocyte counts were performed in the peritoneum and blood of infected or LPS inoculated mice as described previously [3].

### Infections and bacterial counts in macrophages and epithelial cells

Human cervix carcinoma cells (HeLa; American Type Culture Collection No. CCL-2) were grown at 37°C under 5% CO_2_ in Eagle's minimal essential medium supplemented with 5% fetal bovine serum, 2.5% sodium bicarbonate and 1% glutamine (all from Gibco, Inc.). Murine macrophages (Raw 264.7; American Type Culture Collection No. TIB-71) were grown at 37°C under 5% CO_2_ in Dulbecco's medium supplemented with 10% fetal bovine serum, 2.5% sodium bicarbonate and 1% glutamine (all from Gibco). Penicillin (100 units/ml) and streptomycin (100 µg/ml) routinely added, were excluded from cell cultures during *Ochrobactrum* and *Brucella* infections. Before infection, 24-well-plates monolayers were washed in PBS and kept at 4°C. Infections were carried out using cultures of *O. anthropi* or *B. abortus* in logarithmic phase diluted in Eagle's minimal essential medium to reach the desired multiplicities of infection (MOI). Plates were centrifuged at 300×g at 4°C, incubated for 30 min at 37°C under 5% CO_2_ and washed 3 times with PBS. Extracellular bacteria were killed by adding Eagle's minimal essential medium supplemented with 100 µg/ml gentamicin for 1 h for *B. abortus* or 4 h for *O. anthropi*. Cells were further incubated for the indicated times in the presence of 5 µg/ml gentamicin for *B. abortus* or 100 µg/ml gentamicin for *O. anthropi*. Plates were then washed with PBS. Cells were lyzed with 0.1% Triton X-100 for 10 min. Aliquots were diluted and plated in tryptic soy agar and incubated at 37°C for determination of CFU. For immunofluorescence experiments HeLa cells or Raw 264.7 macrophages grown on 12 mm glass slides were inoculated with *B. abortus* or *O. anthropi* as described above and kept in the presence of gentamicin for the indicated times. After incubation cells were fixed with 3.7% paraformaldehyde-PBS and free aldehyde groups were quenched with 50 mM NH_4_Cl-PBS. After permeabilization with 0.5% Triton-PBS, total bacteria were labeled with rabbit anti-*Ochrobactrum* antibodies followed by TRITC conjugated anti-rabbit antibodies.

### Confocal microscopy and flow cytometry of LPS treated dendritic cells

Immunofluorescence confocal microscopy and flow cytometry of LPS treated murine bone marrow derived dendritic cells (BMDC) were performed as described before [72]. Briefly, paraformaldehyde fixed cells processed by immunofluorescence were examined on a Zeiss LSM 510 laser scanning confocal microscope for image acquisition. Antibody against a conserved cytoplasmic epitope found on MHC-II I-Aβ which strongly labels BMDC but not macrophages was used throughout the experiments. For confirmation of the dendritic cell phenotype, double labeling with anti- MHC-II and anti-CD11c antibody was performed. Quantification was always done by counting at least 100 cells in 4 independent experiments, for a total of at least 400 host cells analyzed. DALIS BMDC were collected and stained immediately before fixation. Isotype controls were included as well as BMDC non-LPS treated as control for autofluorescence. Cells were always gated on CD11c for analysis and at least 100000 events were collected to obtain a minimum of 10000 CD11c positive events for analysis. A FACS calibur cytometer (Becton Dickinson) was used and data was analyzed using FlowJo software (Tree Star).

### Electrophoretic and immunochemical analysis

The different bacterial strains were grown to exponential phase in TSB at 37°C. The bacteria were concentrated by centrifugation (10000×g, 10 min), resuspended in Laemmli sample buffer [75] and heated at 100°C for 20 min. Protein concentration was determined by BioRad DC method according to the manufacturer's instructions and equal amounts of protein (20 µg) were loaded onto a 12.5% gel for SDS-PAGE. Separated proteins were transferred to PVDF membrane and probed with the indicated antibodies. Membranes were further incubated with peroxidase conjugated anti-mouse or anti-rabbit antibodies and the detected bands were visualized by chemiluminescence reaction exposed to X-ray films.

### PCR and RT-PCR

Genomic DNA was isolated by CTAB method according to standard procedures [76]. Specific amplification from gDNA or cDNA was achieved with the following primers designed after analysis of *O. anthropi* genome: for locus Oant_0677: oantvirB4f GGATTACACCGTGACCTCAAC and oantvirB4r GCCTGATAACATGCGTCCATAA; for locus Oant_0678: oantvirB5.3 CGATGCTCTTCATCAGCAGATTGAG, oantvirB5.5 CTTTGCGACATCCGCCATATC; for locus Oant_0682: oantvirB8.3 GATCAAGACCGCATGATTCAGG, oantvirB8.5 GTCACGGCTTCGTCGTAAGTG. For amplification of gDNA or cDNA encoding putative *bvrR* and *bvrS*, the following primers were used: for Oant_0827 (*bvrS*): oantbvrS.3 GCATTCTCTACATGAATCAGTTCC and oantbvrS.5 GTGATGGCGTCAGGCTTTG. And for Oant_0828 (*bvrR*): oantbvrR.3 GACGACGACCGCAACATCCTG and oantbvrR.5 CGAAGATCGCAAGATTGGGC. Positive controls of amplification of genomic DNA or cDNA were achieved using a pair of primers oantl12.3 CTGTCGGCACTGACCGTTCTCG and oantl12.5 CGCCACCAGCAGCAGCAACTG interrogating locus Oant_1946, encoding the ribosomal protein L7/L12. Cycling conditions were: 94°C for 4 min, 30 cycles at 94°C for 30 s, 50 or 65°C for 1 minute and 68°C for 30 seconds and continued with a 68°C incubation for 10 minutes. The PCR products were analyzed on agarose gels using standard procedures. For RNA extraction bacterial cultures were adjusted to 10^9^ CFU/ml and disrupted with 0.5% Zwittergent 3–16 at 37°C for 1 h. Total RNA was extracted using RNeasy Midi Kit (Qiagen, Co.) according to the manufacturer's instructions. Eluted RNA was treated with Ambion Turbo DNAse following the robust protocol. Synthesis of cDNA was carried out using Revert Aid M-MuLV Reverse Transcriptase with random hexamers and following manufacturers' Instructions. (Fermentas). The obtained cDNA was used as template for detection of the genes mentioned above by PCR using the same primers described. Minus RT controls were performed using RNA samples during PCR.

### Complement depletion and bactericidal action of serum

Complement consumption was estimated as previously described [77], [78]. Briefly, rabbit serum was incubated with live packed bacteria or LPS for 30 min at 37°C. After incubation, the complement activity on the serum was detected as the ability to lyze sheep erythrocytes pre-sensitized with guinea pig anti-sheep antibodies. For the estimation of complement bactericidal activity, exponentially growing bacteria were adjusted to 10^4^ CFU/ml in saline and dispensed in triplicate in microtiter plates (45 µl per well) containing fresh normal bovine serum (90 µl/well). After 90 min of incubation at 37°C, brain heart infusion broth was dispensed (200 µl/well), mixed with the bacterial suspension and 100 µl was plated on TSA. Results were expressed as the percentage of CFU with respect to the inoculums in three assays.

### Binding of LPS to hTLR4 co-receptor hMD-2 protein

Binding of LPS to MD-2 was assayed by two different methods: displacement of bis-ANS from hMD-2 by LPS, and binding to hMD-2 by competitive enzyme linked-immunosorbent assay (ELISA). Recombinant hMD-2 was produced in *E. coli* and isolated as described before [79]. In both assays, LPS was subjected to three cycles of heating at 56°C followed by cooling to 4°C, left over night and sonicated before using. Binding of bis-ANS to hMD-2 was measured at 20°C using excitation at 385 nm and measuring the emission fluorescence spectra between 420 and 550 nm. Then, from 5 to 80 µl of a LPS stock at 0.125 mg/ml was added (controls were prepared with the same volume of water) to preincubated bis-ANS/hMD-2 complex (50 nM/50 nM). The F_0_ value was the fluorescence of bis-ANS/hMD-2 complexes after 5 min of incubation (to reach stable fluorescence).The F_LPS_ value was the fluorescence after LPS addition to the complex. Fluorescence was measured on Perkin Elmer fluorimeter LS 55. Quartz glass cuvettes (5×5 and 10×5 mm optical path, Hellma Suprasil) were used and bis-ANS was obtained from Sigma (St. Louis, Missouri, U.S.A.). The ELISA for determination of LPS binding to hMD-2 was performed in 96-well plates (NUNC immunoplate F96 cert. Maxi-sorp, Roskilde, Denmark). Chicken polyclonal anti-hMD-2 (GenTel, Madison, WI, U.S.A) (5 µg/ml) in 50 mM Na_2_CO_3_ (pH 9.6) was used to coat the microtiter plate at 4°C overnight. Excess binding sites were blocked with 1% BSA in PBS buffer (pH 7.2) for 1 h at room temperature, and rinsed three times with the same buffer. During the blocking step, hMD-2 (0.75 µM) was preincubated with 0 µg/ml to 20 µg/ml LPS at 37°C and, as a negative control, LPS was also preincubated in absence of hMD-2. These solutions were added to the plate, which was then incubated for 1 h at 37°C. After rinsing, only hMD-2 without LPS was detected by incubation with 0.1 µg/ml of mouse monoclonal anti-hMD-2 (clone 9B4 e-Bioscience San Diego, CA., U.S.A.) in PBS buffer at 37°C for 1 h, followed by incubation with 0.1 µg/ml peroxidase-conjugated goat anti-mouse IgG (Santa Cruz, CA., U.S.A.), also in PBS buffer at 37°C for 1 h. After plate washing, ABTS (Sigma) was added, the reaction was stopped with 1% SDS after 15 min, and the absorbance at 420 nm measured using a Mithras LB940 apparatus. (Berthold Technologies). *S. enterica* (strainHL83) LPS, used as a control.

Stimulation of NF-κB transcription by purified LPS. HEK293 cells (ATCC CRL-1573) were transiently transfected with 3 ng hTLR4, 6 ng hMD-2, 5 ng hCD14 expression vectors, 50 ng of NF-κB or IP-10 promoter dependent luciferase reporter, and 5 ng constitutive Renilla reporter plasmids, using lipofectamine 2000 (Invitrogen). After 6 h, media were changed to Dulbeccos's modified Eagle's medium supplemented with 10% fetal bovine serum and different amounts of LPS were added to the cells. As a negative control, we used cells transfected with the same plasmids but without addition of LPS. After 20 h, cells were lyzed and analyzed for reporter gene activities using a dual luciferase reporter assay system on a Mithras LB940 apparatus (Berthold Technologies). The data of luciferase activity were normalized using Renilla luciferase readings.

### Statistical methods

Student's t test for was used for determining the statistical significance in the different assays. For bacterial colonization experiments, the Mann-Whitney test was performed accordingly (http://faculty.vassar.edu/lowry/utest.html).
